# A Guide to Diseases of Children

**Published:** 1885-10

**Authors:** M. P. Hatfield


### Book Reviews.

A Guide to the Diseases of Children. *By* James Frederick Goodhart, m. d., f. r. c. p., etc. *Revised and edited by* Louis Starr, m. d. *Philadelphia:* P. Blakiston, Son & Co. *Chicago:* W. T. Keener. Pp. 728.

The careful reading of this book will convince any one interested in pædiatrics that Dr. Goodhart has admirably succeeded in preparing a manual for ready reference. No one but a German specialist, or a physician of the days of Methuselah, has time to read, mark and inwardly digest such a work as Gerhardt's *Handbuch der Kinderkrankheiten.* In a lesser degree a similar desire to make their efforts encyclopædic has unduly expanded all of the more recent text books on children's diseases. Dr. Goodhart has avoided this by omitting much useless history and bibliography, giving instead especial prominence to the differentiation and essential differences observed in disease in children as compared with like affections in the adult. "At first all our cases are phthisical; a riper experience shows them to be extremely rare," is a sample of the honesty and acknowledgment of practical difficulties that pervades the entire work. In fact, it reads from beginning to end like the kindly advice of an elder to a younger practitioner. There is nothing of the "I myself have said it" in this; but, omitting all that can be fairly granted ought to be known by the general practitioner in the way of histology and semiology, the writer clearly states wherein he has found disease in childhood other than what might have been expected, either in symptoms, course or results of treatment. It is a confidential professional talk—not unlike Dr.

Jackson's "Letters to a Young Physician," now unfortunately out of print—by one who is not striving to pad out a book, but who really has something to add to our common store of knowledge from years of careful observation. It is just such a book as ought to be the outcome from every well-equipped hospital; not a mass of dreary, dry statistics, but practical, hopeful hints for the better treatment of disease by those who cannot otherwise share hospital advantages. This, if we mistake not, has been Dr. Goodhart's aim, and most excellently has he accomplished it. He has given us no beautiful theories or false hopes founded upon hypothetical methods of treatment, but instead, plain practical, helpful suggestions for the better recovery of sick children. He has evidently given a fair trial to the various methods of treatment, and approves or disapproves according to results, and not in accordance with their present popularity, a very good instance of which is the following extract concerning the early operation of tracheotomy: "Now I say that in diphtheria it is a serious operation, * * * and as a matter of fact, the operation of tracheotomy, when performed upon the diphtheric child, is frequently followed by diffuse inflammation of the cellular tissue of the neck, and a large, sloughy wound is formed, * * whilst it would be easy to show in the clearest manner, in the *post-mortem* room, that the operation itself and the presence of the tube afterwards are in one way and another fraught with danger. It is, in fact, my belief that the brocho-pneumonia and purulent bronchitis and excessive tracheitis so often seen are chargeable quite as often to the operation as to the original disease."

Dr. Starr's notes are always timely and valuable to the American reader. Mechanically, the book is in every way a credit to its makers.

M. P. HATFIELD.

